# Editorial: Plant growth-promoting and associated microbes: multi-, meta-omics, and applications

**DOI:** 10.3389/fgene.2024.1414750

**Published:** 2024-05-20

**Authors:** Mahloro Hope Serepa-Dlamini, Ntakadzeni Edwin Madala, Matsobane Godfrey Tlou

**Affiliations:** ^1^ Department of Biotechnology and Food Technology, Faculty of Science, University of Johannesburg, Johannesburg, South Africa; ^2^ Department of Biochemistry and Microbiology, Faculty of Science, Engineering and Agriculture, University of Venda, Thohoyandou, South Africa; ^3^ School of Physical and Chemical Sciences, Department of Biochemistry, North-West University, Mmabatho, South Africa

**Keywords:** multiomics, metaomics, plant microbiome, plant growth promotion, secondary metabolism, GCMS, biological activity

The application of multiomics, metaomics, and data analyses has allowed the identification and characterization of plant-growth-promoting microbes, including endophytes. Plants live in association with various beneficial microbes, such as endophytes, which are important for host growth, development, and health. Despite the availability of many reports on endophytes, very few studies have evaluated the complex network of genetic, biochemical, physical, and metabolic interactions between plants and their associated microorganisms, especially for woody, forest, and desert plants. This delineates the possible functions of the plant microbiome, which can be adopted for application to various sectors.

The Research Topic “*Plant-growth-promoting and associated microbes: multiomics, metaomics, and applications*” showcases some of the multiomics and metaomics techniques used to describe the functions of endophytes in plant growth and development, thus highlighting possible applications of endophytes in various sectors, such as agriculture, medicine, and drug discovery.

This Research Topic includes one review article by Zamanzadeh-Nasrabadi et al. highlighting the significant impact of salinity stress on agricultural productivity and the role of halotolerant bacterial endophytes in mitigating this stress; this work emphasizes the importance of halotolerant bacterial endophytes, particularly those belonging to the Firmicutes, Proteobacteria, and Actinobacteria phyla, in alleviating salinity stress. These bacteria are rich in several genes related to various mechanisms, such as the synthesis of indole acetic acid, siderophores, osmoprotectants, chaperones, ACC deaminase, and antioxidants as well as phosphate solubilization and ion homeostasis, further demonstrating their ability as endophytes in plant growth and protection.

There are also research articles reporting on the various secondary metabolites produced by bacterial and fungal endophytes that not only play roles in plant growth and protection but also have potential applications in the treatment of human immunodeficiency virus (HIV) and multidrug-resistant fungi. Nzimande et al. explore the potential of secondary metabolites produced by endophytic fungi through gas chromatography mass spectrometry (GCMS), specifically *Alternaria alternata*, as inhibitors of HIV. They discuss how various fungal secondary metabolites have been observed to inhibit different stages of the HIV-1 life cycle using examples like cyclotrisiloxane octamethyl; propaninitrile; pyrrolo[1,2-a]pyrazine-1,4-dione, hexahydro-3-(2-methylpropyl); silane, diethylethoxy (2-ethoxyethyloxy); coumarin, 3,4-dihydro-4,5,7-trimethyl-4,5,7-trimethyl-2-chromanone; and 1,2-cyclobutanedicarbonitrile that have also shown anti-inflammatory and antioxidant properties.

Sui et al. investigated the role of the *Beauveria bassiana* endophyte in enhancing plant growth and resistance to pathogens. They highlighted how *B. bassiana* colonizes tomato plants under *Botrytis cinerea* infection stress and its impact on the disease resistance of the plant by collecting the pathogen-infected leaves, inducing the expression of plant resistance genes, and enhancing the plant growth and resistance against pathogens.

Understanding the roles of endophytes in the plant–endophyte interactions also calls for understanding the plant physiology, structures, and responses to infection by pathogens or endophytes. Zhang et al. used transcriptomics to delve into the molecular responses of pepper plants to pepper mild mottle virus (PMMoV) infection. Their study focused on two pepper genotypes, one tolerant (17-p63) and one susceptible (16-217), to understand their responses to PMMoV at the transcriptome level, and the results indicated that the resistant genotype 17-p63 exhibited lower viral accumulation and milder infection symptoms than the susceptible genotype 16-217. This study identified the genes implicated in the disease or pathogen responses, thus demonstrating the molecular mechanisms of plant resistance to pathogen infection.

By integrating multiomics data, we can unravel the complex dynamics of plant–endophyte interactions, including the impacts of microbial communities on crop resilience, nutrient uptake, and defense mechanisms. These insights are crucial for developing sustainable agricultural practices, reducing chemical inputs, and harnessing the potential of plant-associated microbiota to enhance crop productivity while mitigating the effects of abiotic and biotic stresses, in addition to harnessing the endophytes for applications in various fields. Although minimal, this Research Topic highlights the versatility of endophytes and the benefits of using omics-based approaches to studying and understanding plant–endophyte interactions.

